# Ultrasound-guided transversus abdominis plane block in cesarean delivery: a randomized trial of ketamine versus neostigmine as bupivacaine adjuvants

**DOI:** 10.1186/s44158-026-00371-1

**Published:** 2026-03-25

**Authors:** Ayman Mohamed Maaly, Hussien Mohamed Agameya, Mohamed Mohamed ElNakeeb, Steven Naser Abdul-Malak

**Affiliations:** Department of Anaesthesia and Surgical Intensive Care, Faculty of Medicine, Alexandria University, Alexandria, Egypt

**Keywords:** Ketamine, Neostigmine, Transversus abdominis plane block, Cesarean delivery

## Abstract

**Background:**

Cesarean delivery (CD) stands as one of the most prevalent surgical procedures for childbirth. Ensuring effective pain management during and after CD is paramount to safeguarding the well-being and comfort of the mother. The transversus abdominis plane (TAP) block is a widely used technique for postoperative pain control. To enhance the duration and quality of analgesia provided by TAP blocks, adjuvants such as ketamine and neostigmine have been investigated.

**Methods:**

A prospective, double-blind randomized controlled study was adopted. Eighty patients scheduled for elective CS were randomized into two equal groups after receiving spinal anesthesia. Group K received postoperative TAP block with plain bupivacaine and ketamine and Group N received TAP block with bupivacaine and neostigmine.

**Results:**

A significantly higher mean arterial blood pressure was observed at 18 h and 24 h in Group N compared to Group K. Comparing the postoperative visual analogue scale score, no significant differences were observed at 2 h, 4 h, and 6 h. At 12 h post-procedure, the mean VAS score was significantly higher for Group N compared to Group K. This trend continued at 18 and 24 h (*p* < 0.001).

**Conclusion:**

Both adjuvants effectively controlled postoperative pain, with stable intraoperative hemodynamics and high patient satisfaction levels. Ketamine demonstrated superior analgesic efficacy compared with neostigmine in cesarean delivery patients undergoing TAP blocks. An individualized treatment approach is needed to tailor the selection of adjuvants to optimize outcomes.

## Introduction

Cesarean delivery (CD) is one of the most commonly performed surgeries for childbirth. Managing pain effectively during and after the procedure is essential for the mother’s comfort and overall health. Good pain control is a key part of obstetric care, as it influences not only the surgical experience but also the mother’s recovery and satisfaction. Because CD is a complex procedure and there is a growing effort to reduce the use of systemic painkillers, regional anesthesia methods have been increasingly explored. One such method, the transversus abdominis plane (TAP) block, has become well known for its ability to offer focused and long-lasting pain relief [1].

The transversus abdominis plane (TAP) block is a commonly used method for controlling pain after surgery. It works by injecting local anesthetics into the space between the internal oblique and transversus abdominis muscles [2]. Ketamine and neostigmine have been investigated as potential additives to local anesthetics in regional anesthesia to enhance analgesic duration and reduce opioid requirements [3].

Ketamine is believed to exert peripheral analgesic effects through N-methyl-D-aspartate (NMDA) receptor antagonism and modulation of inflammatory pathways [4]. Neostigmine, an acetylcholinesterase inhibitor, may increase acetylcholine levels in peripheral tissues, potentially activating muscarinic receptors and enhancing peripheral analgesic effects [5].

In recent years, both ketamine and neostigmine have attracted interest as additives to plain bupivacaine in ultrasound-guided TAP blocks for cesarean delivery. These agents have shown encouraging results in prolonging postoperative pain relief, lowering opioid use, and boosting patient satisfaction. Nonetheless, there is still ongoing discussion regarding their relative effectiveness and safety [6].

The present study aimed to evaluate whether adding low-dose ketamine or neostigmine to bupivacaine in TAP blocks could improve postoperative analgesia and reduce opioid consumption following cesarean delivery. The goal is to provide clinicians with evidence-based guidance on selecting the most suitable adjuvant by evaluating its effectiveness, safety, and impact on patient outcomes. The findings of this study may contribute to the development of standardized protocols and guidelines for TAP block administration in elective CD, ultimately improving postoperative pain control and enhancing patient recovery.

## Materials and methods

This study was registered at ClinicalTrials.gov (NCT06871033 on 3/3/2025) before the start of the trial and any patient enrollment undertaken. This manuscript adheres to the applicable CONSORT guidelines.

The present study was carried out in a University Hospital on 80 patients scheduled for elective CD. The sample size was calculated using Power Analysis and Sample Size Software (PASS 2020). The primary outcome for the sample size calculation was the postoperative Visual Analogue Scale (VAS) score at 12 h. Based on prior studies and clinical relevance, we considered a clinically meaningful difference of 1.9 points (SD ≈ 3.0), corresponding to an effect size of approximately 0.63. Using a two-tailed *α* = 0.05 and 80% power, the calculated sample size was 40 patients per group. Thus, a total of 80 patients were enrolled [7].

ASA physical status II multiparous women undergoing elective CD were included in the study. On the other hand, patients undergoing emergent CD, those who had a prolonged surgery lasting more than 120 min [8], and those who had an allergy to local anesthesia or any additive drug, coagulopathy, peripheral neuropathy, chronic pain syndrome, localized infection, body mass index > 35, or preeclamptic and eclamptic pregnant females were excluded. Only multiparous women were included to reduce variability in pain perception.

A prospective, double-blind randomized controlled study was adopted. An assistant nurse was assigned and taught to prepare the injectable drugs to ensure the double blindness of the study. Then, every patient was recorded to the assigned syringes and revised with the nurse.

Patients were randomly allocated into two equal groups using a computer-generated randomization list (permuted blocks of size 4). Allocation assignments were kept in sequentially numbered, opaque, sealed envelopes prepared by an independent person not involved in the study. A nurse not involved in data collection prepared the study syringes. Both patients and assessors were blinded to group allocation.

After receiving spinal anesthesia and completion of CD surgery:

Group 1 (K): 40 patients received postoperative TAP block with 20 ml 0.25% plain bupivacaine (10 ml 0.5% bupivacaine added to 10 ml normal saline) and 0.5 mg/kg ketamine for each side [9].

Group 2 (N): 40 patients received postoperative TAP block with 20 ml 0.25% plain bupivacaine (10 ml 0.5% bupivacaine added to 10 ml normal saline) and 0.5 mg neostigmine for each side [10].

### Preoperative assessment and preparation

Proper history taking, complete clinical examination, and routine laboratory investigations were done. The technique of anesthesia and visual analog score (VAS) were explained to patients. The VAS score is a simple and commonly used method to measure pain intensity. It involves a horizontal line, usually 10 cm long, with one end representing “no pain” and the other end representing “worst pain imaginable.” Patients were asked to mark a point on the line that corresponds to their current pain level. This helped understand the severity of pain and determine the appropriate pain management strategies [11].

The difference between somatic or parietal pain and visceral pain was explained to patients. Somatic or parietal pain arises from the outer layers of the body, such as the skin, muscles, and tissues. It is typically well-localized, sharp, and increases with movement. In contrast, visceral pain originates from internal organs and their coverings and is often described as dull, aching, or cramp-like that does not increase with movement. By understanding these distinctions, patients can better communicate their pain experiences.

### Patient preparation

The patients received oral Famotidine 40 mg at night before the day of surgery and ondansetron 4 mg IV immediately before surgery.

### Monitoring

A multichannel monitor (Dräger, Vista 120) was connected to the patient to display continuous electrocardiography monitoring for heart rate (beat/min) and detection of dysrhythmias, noninvasive arterial blood pressure (NIBP), and pulse oxygen saturation (SpO2).

### Anesthetic technique

All patients undergoing elective CD surgery received spinal anesthesia as the primary anesthetic technique. Spinal anesthesia was performed at the L3–L4 or L4–L5 interspace using hyperbaric bupivacaine. The intrathecal dose was adjusted according to patient height, as per institutional routine practice (< 150 cm: 9 mg, 150–160 cm: 10 mg, 160–170 cm: 11 mg, > 170 cm: 12 mg). No intrathecal opioids were administered. This reflects our institutional protocol and was intentionally chosen to allow clearer evaluation of the postoperative analgesic effect of the TAP block and its adjuvants without confounding prolonged spinal opioid analgesia. Following spinal injection, dermatomal sensory assessment was performed using loss of pinprick sensation. Surgery was allowed to proceed only after achieving a sensory block up to the T4 dermatome (nipple line).

All procedures were performed according to institutional standardized protocol. Following the completion of the CD surgery, a transversus abdominis plane block was performed to enhance postoperative pain management. Strict sterile techniques were followed.

The TAP block was carried out using a Sonosite M-Turbo ultrasound machine with a high-frequency (10–15 MHz) linear probe. The transducer was positioned in the axial plane along the midaxillary line, between the subcostal margin and the iliac crest. The three layers of the abdominal wall muscles—external oblique, internal oblique, and transversus abdominis—were identified. The target area was the fascial plane between the internal oblique and transversus abdominis muscles. A needle was inserted at the anterior axillary line and advanced until it reached the target plane. In the first group, 20 ml of 0.25% plain bupivacaine combined with 0.5 mg/kg ketamine was injected. In the second group, 20 ml of 0.25% plain bupivacaine with 500 µg of neostigmine was used. Injections were done under ultrasound guidance with aspiration after every 5 ml to ensure safety, and successful separation of the plane was confirmed by increased hypoechoic separation of the fascial plane around the midaxillary line. The same procedure was repeated on the opposite side.

All patients received intravenous paracetamol (1 g every 8 h for 24 h) as baseline non-opioid analgesia. Rescue analgesia consisted of intravenous nalbuphine administered as needed. No additional opioids were used.

### Measurements

The following parameters were measured in every patient in the study:

1. Vital signs including heart rate (bpm) and mean arterial blood pressure (mmHg). Baseline blood pressure and heart rate were recorded preoperatively before the administration of spinal anesthesia. These parameters were continuously monitored and recorded intraoperatively and postoperatively after giving TAP block with adjuvants, at 2, 4, 6, 12, 18, and 24 h postoperative.
2. Pain scoring where the visual analog scale was recorded intraoperatively and at 2 h, 4 h, 6 h, 12 h, 18 h, and 24 h postoperatively. The rescue analgesia (nalbuphine 6 mg IV) was used when VAS score ≥ 4.
3. Duration of analgesia (hours) defined as the time interval after the start of the technique till the first need for rescue analgesic (nalbuphine 6 mg IV).
4. Total dose of rescue analgesia (mg), i.e., the cumulative dose of nalbuphine (mg) administered during the first 24 postoperative hours, including repeated doses if required.
5. Patient satisfaction with pain control assessed on a four-point scale: Excellent, Good, Fair, and Poor.
6. Postoperative complications within the 24 h of the study (e.g., hypotension or hematoma at the site of injection) were recorded and analyzed accordingly.

### Statistical analysis

Data was fed to the computer and analyzed using IBM SPSS software package version 23.0. (Armonk, NY: IBM Corp). The Shapiro–Wilk test was used to verify the normality of distribution. Continuous variables are expressed as mean ± SD and compared using the independent samples *t*-test (Welch correction applied when variances were unequal). Mann-Whitney *U* test is used to compare non-normally distributed variables. Categorical variables were compared using the chi-square (*χ*^2^) test. All tests were two-tailed, and a *p*-value < 0.05 was considered statistically significant.

## Results

**Fig. 1:**
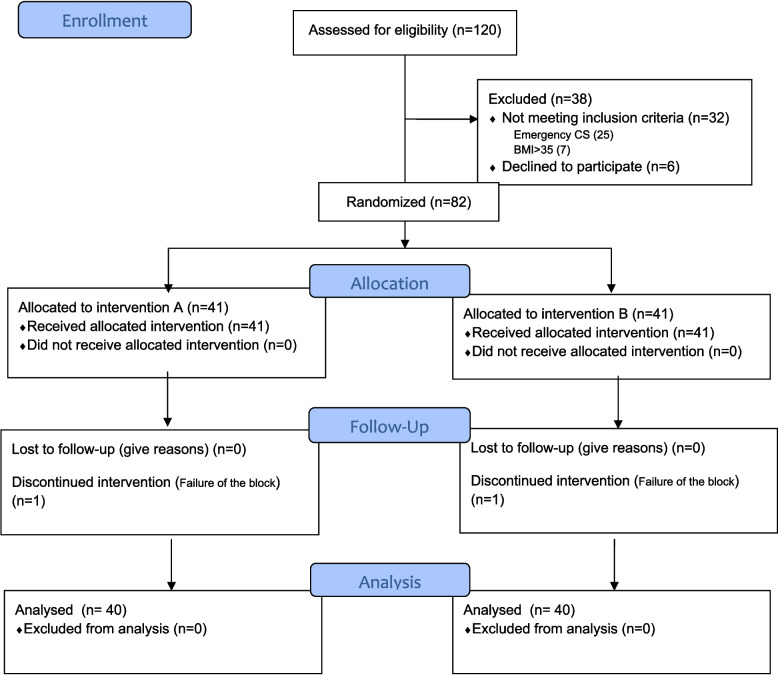
CONSORT 2010 flow diagram of the study. Flowchart illustrating patient enrollment, allocation, follow-up, and analysis among the two study groups

A total of 80 patients completed the study (40 per group). The CONSORT flow diagram of the study is presented in Fig. 1. It illustrates patient enrollment, allocation, follow-up, and analysis among the two study groups.

There were no significant differences in baseline demographics between the two groups (Table 1).

**Table 1:** Comparison between the two studied groups according to age, body mass index, and duration of surgery

Studied variables	Group K	Group N	*t*	*p*
**Age (years)**			***t* = 1.866**	**0.067**
Min.–max.	26–41	27–42		
Mean ± SD	35.15 ± 3.61	33.50 ± 4.27		
**Body mass index (kg/m**^**2**^**)**			***t* = 1.763**	**0.082**
Min.–max	29–35	28–35		
Mean ± SD	31.75 ± 1.98	30.85 ± 2.55		
**Duration of surgery (minutes)**			***t* = − 0.237**	**0.813**
Min.–max	62–118	60–119		
Mean ± SD	91.93 ± 12.50	92.75 ± 18.11		

*t*: Student’s *t* test

As illustrated in Tables 2 and 3, heart rate and mean arterial blood pressure (MABP) were comparable between groups from baseline through 12 h postoperative (*p* > 0.05 for all comparisons). At 18 and 24 h, the ketamine group showed significantly lower heart rate and MABP compared with the neostigmine group (*p* < 0.05).

**Table 2:** Comparison between the two studied groups regarding heart rate (beats/min)

Mean heart rate (bpm)	Group K	Group N	*Z*	*p*
Preoperative	91.57 ± 11.50	88.60 ± 11.19	− 1.093	0.274
Intraoperative	102.80 ± 10.77	99.58 ± 10.47	− 1.339	0.181
Postoperative				
2 h	86.05 ± 11.60	83.20 ± 11.05	− 1.035	0.301
4 h	88.52 ± 11.62	85.80 ± 11.23	− 0.939	0.348
6 h	90.67 ± 11.39	87.60 ± 11.20	− 1.228	0.219
12 h	89.42 ± 11.60	86.67 ± 11.29	− 1.030	0.303
18 h	81.73 ± 11.36	91.60 ± 11.11	− 3.067	0.002*
24 h	79.75 ± 11.49	96.85 ± 11.10	− 5.334	< 0.001*

*Z*: value of Mann–Whitney *U* test

^*^Significant at *p* < 0.05

**Table 3:** Comparison between the two studied groups regarding mean arterial blood pressure (mmHg)

MABP (mmHg)	Group K	Group N	*Z*	*p*
Preoperative	84.38 ± 8.58	81.65 ± 8.58	− 1.378	0.168
Intraoperative	78.68 ± 8.86	76.10 ± 8.80	− 1.233	0.218
Postoperative				
2 h	80.40 ± 8.61	77.70 ± 8.39	− 1.363	0.173
4 h	83.35 ± 8.54	80.65 ± 8.58	− 0.397	0.162
6 h	85.43 ± 8.55	82.93 ± 8.65	− 1.306	0.192
12 h	88.85 ± 8.52	85.70 ± 8.54	− 1.652	0.099
18 h	82.15 ± 8.51	87.75 ± 8.58	− 2.379	0.017*
24 h	79.35 ± 8.54	89.80 ± 8.54	− 4.537	< 0.001*

*Z*: value of Mann–Whitney *U* test

^*^Significant at *p* < 0.05

**Fig. 2:**
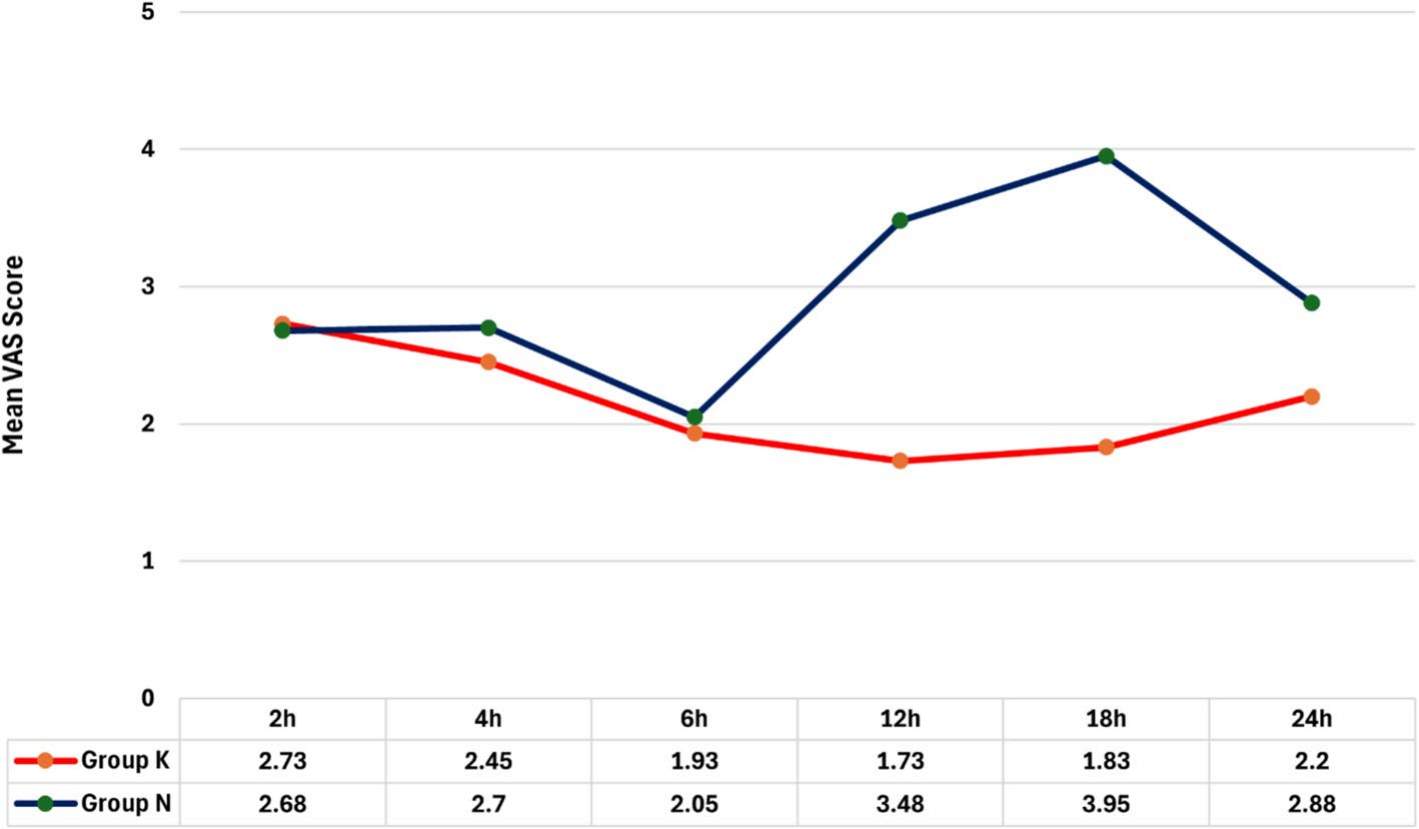
Postoperative visual analogue scale (VAS) pain scores in the two study groups. Line graph showing VAS scores measured at 2, 4, 6, 12, 18, and 24 h after cesarean delivery in patients receiving transversus abdominis plane (TAP) block with ketamine (Group K) or neostigmine (Group N) as adjuvants to bupivacaine

Comparing the postoperative visual analogue scale score between the two groups, Fig. 2 illustrates that no significant differences were observed at 2 h, 4 h, and 6 h. At 12 h post-procedure, Group K had a significantly lower mean VAS score (*p* < 0.001). This trend continued at 18 h (*p* < 0.001) and at 24 h (*p* = 0.012).

The duration of analgesia was significantly longer in the ketamine group (20.58 ± 2.70 h) compared with the neostigmine group (16.08 ± 4.12 h; *t* = 5.783; *p* < 0.001) and the total rescue analgesic dose was significantly lower in the ketamine group (3.90 ± 4.99 mg) than in the neostigmine group (9.90 ± 5.85 mg; *t* = −4.929; *p* < 0.001).

Patient satisfaction levels, as shown in Table 4, were comparable between the groups (*χ*^2^ = 1.429, *p* = 0.698). Both groups displayed relatively high levels of satisfaction, with most patients rating their experience as either Excellent or Good.

**Table 4:** Comparison between the two studied groups regarding patient satisfaction

Patient satisfaction	Group K (*n* = 40)	Group N (*n* = 40)	*x*^2^	^MC^*p*
Excellent	21 (52.5)	20 (55 = 0.0)	1.429	0.698
Good	12 (30.0)	16 (40.0)		
Fair	5 (12.5)	3 (7.5)		
Poor	2 (5.0)	1 (2.5)		

*x*^2^: chi-square (Monte Carlo) test

## Discussion

The present study demonstrated that the addition of ketamine resulted in superior postoperative analgesia compared with neostigmine, as evidenced by significantly lower VAS scores from 12 to 24 h, prolonged duration of analgesia, and reduced rescue analgesic consumption. These findings contribute to the optimization of pain management strategies in obstetric anesthesia, ultimately improving patient outcomes and satisfaction in the perioperative period.

Both groups maintained stable hemodynamic parameters intraoperatively and postoperatively. The ketamine group had a significantly lower mean postoperative HR and MABP than the neostigmine group after 18 and 24 h. Possible explanations for the observed hemodynamic variability between the two groups could relate to the pharmacological properties and mechanisms of action of ketamine and neostigmine. Ketamine, as an NMDA receptor antagonist, may exert sympathomimetic effects, potentially contributing to the maintenance of stable heart rates and MABP after surgery. In contrast, neostigmine’s parasympathomimetic effects, resulting from acetylcholinesterase inhibition, may lead to more variable hemodynamic responses, including fluctuations in heart rate and blood pressure.

These results are supported by Abdelhamid BM et al. [7] who found that ketamine and neostigmine, when used as adjuvants in regional anesthesia, did not compromise hemodynamic stability. They found that adding 50 mg ketamine to bupivacaine in Serratus Anterior Plane Block significantly reduced 24-h postoperative morphine consumption and intraoperative fentanyl use in patients undergoing modified radical mastectomy, while 500 µg neostigmine reduced only intraoperative fentanyl use. Moreover, Bouderka MA et al. [10] who demonstrated that adding 500 µg neostigmine to bupivacaine in axillary plexus block significantly reduced postoperative pain scores (VAS) and ketoprofen consumption without increasing side effects. This combination effectively prolonged postoperative analgesia. In addition, individual patient characteristics, such as baseline cardiovascular status, comorbidities, and medication history, could influence hemodynamic responses to ketamine and neostigmine. Variability in anesthesia management practices, including fluid administration, vasopressor use, and surgical techniques, may also be a contributing factor.

The present study found that both ketamine and neostigmine effectively managed postoperative pain following cesarean delivery, as evidenced by decreasing mean VAS scores with minimal variability among patients in each group. The ketamine group showed significantly lower mean VAS scores compared to the neostigmine group in the postoperative period, indicating better pain control. The differences in pain perception may be attributed to the distinct pharmacological actions of ketamine’s NMDA receptor antagonism and neostigmine’s acetylcholinesterase inhibition. Individual patient factors, such as baseline pain thresholds and comorbidities, may also have influenced variations in pain experiences.

These findings are in agreement with Edinoff et al. [12] who reported enhanced postoperative analgesia when ketamine was used as an adjuvant. In addition, Abdelhamid BM et al. [7] also reported superior analgesic efficacy of ketamine in reducing morphine consumption when it is used as an adjuvant to bupivacaine in regional blocks. However, Habib et al. [13] demonstrated that neostigmine’s cholinesterase inhibition amplifies local anesthetic action, corroborating its efficacy in reducing pain scores. Also, Saleh et al. [14] concluded that both agents extend analgesia without a clear superiority. The authors compared ketamine and neostigmine as adjuvants to bupivacaine in ultrasound-guided serratus anterior plane block for patients undergoing modified radical mastectomy. Ketamine significantly reduced 24-h nalbuphine consumption and postoperative pain scores compared to saline, while neostigmine also lowered rescue analgesia requests but to a lesser extent than ketamine. Both adjuvants enhanced analgesic outcomes without adverse effects, making them effective options for improving block efficacy.

Contrastingly, Bouderka et al. [10] found inconsistent pain relief with neostigmine in nerve blocks, possibly due to variations in dosage and patient populations. The current findings align more closely with studies where neostigmine’s dosing was optimized.

Regarding patient satisfaction, both groups reported high patient satisfaction levels. This finding aligns with Bhakta et al. [15] who associated effective pain management with enhanced patient satisfaction.

The non-significant difference in satisfaction between groups is in agreement with Mansour et al. [9] who observed that both ketamine and neostigmine improve patient-reported outcomes, though individual perceptions of pain relief can vary. They demonstrated that adding ketamine to levobupivacaine in ultrasound-guided transversus abdominis plane (TAP) block significantly improved postoperative analgesia for abdominoplasty patients. The ketamine group had lower VAS scores, a longer time to first rescue analgesia (18.7 vs. 6.5 h), and reduced morphine consumption compared to levobupivacaine alone, supporting ketamine as an effective adjuvant in TAP blocks.

Regarding safety, the incidence of reported adverse effects was comparable between groups. No clinically significant maternal adverse effects were observed during the study period.

This study has several limitations. It included only multiparous women, which may limit generalizability to primiparous patients. Intrathecal opioids and NSAIDs were not used, which may limit comparison with guideline-based multimodal analgesia protocols. Maternal functional outcomes such as early mobilization and breastfeeding quality were not evaluated. Plasma drug levels were not measured; therefore, systemic absorption of the adjuvants cannot be fully quantified. Additionally, the absence of a placebo control group and the single-center design may limit external validity.

## Conclusion

In conclusion, both adjuvants demonstrated effectiveness in providing postoperative pain control, with stable hemodynamics and high patient satisfaction levels observed in both groups. However, ketamine demonstrated superior analgesic efficacy compared with neostigmine in this study population. An individualized treatment approach is needed to tailor the selection of adjuvants, focusing on using ketamine or neostigmine as additives to bupivacaine in TAP blocks to optimize outcomes. Further studies should focus on the use of ketamine and neostigmine as part of multimodal strategies for TAP blocks specifically in cesarean delivery. Additionally, research should explore their application in other surgical procedures and anesthetic regional techniques, such as quadratus lumborum (QL) blocks, to assess their broader utility in regional anesthesia.

## Data Availability

The datasets used and/or analysed during the current study are available from the corresponding author on reasonable request.
